# Comparison of Sequencing Platforms for Single Nucleotide Variant Calls in a Human Sample

**DOI:** 10.1371/journal.pone.0055089

**Published:** 2013-02-06

**Authors:** Aakrosh Ratan, Webb Miller, Joseph Guillory, Jeremy Stinson, Somasekar Seshagiri, Stephan C. Schuster

**Affiliations:** 1 Center for Comparative Genomics and Bioinformatics, Pennsylvania State University, University Park, Pennsylvania, United States of America; 2 Department of Molecular Biology, Genentech Inc., South San Francisco, California, United States of America; University of California, Irvine, United States of America

## Abstract

Next-generation sequencings platforms coupled with advanced bioinformatic tools enable re-sequencing of the human genome at high-speed and large cost savings. We compare sequencing platforms from Roche/454(GS FLX), Illumina/HiSeq (HiSeq 2000), and Life Technologies/SOLiD (SOLiD 3 ECC) for their ability to identify single nucleotide substitutions in whole genome sequences from the same human sample. We report on significant GC-related bias observed in the data sequenced on Illumina and SOLiD platforms. The differences in the variant calls were investigated with regards to coverage, and sequencing error. Some of the variants called by only one or two of the platforms were experimentally tested using mass spectrometry; a method that is independent of DNA sequencing. We establish several causes why variants remained unreported, specific to each platform. We report the indel called using the three sequencing technologies and from the obtained results we conclude that sequencing human genomes with more than a single platform and multiple libraries is beneficial when high level of accuracy is required.

## Introduction

The Human Genome Project [1], [2] published the first working draft of the human reference sequence in 2000. That sequence was generated in its entirety by capillary sequencing; all subsequent genomes of human individuals [3]–[9], except one [4], have relied on next-generation sequencing (NGS) platforms. Starting in 2005, 454/Roche [10], and subsequently Illumina [11] and SOLiD/ABI [12] entered the market with technology that ultimately aims to re-sequence human genomes for less than $1000, which would transform the field of personalized medicine. While several new entrants may have the future potential to change the sequencing landscape yet again, the current sequencing market is dominated by these three matured platforms. With the introduction of these technologies, reports of biases in all platforms [13], as well as efforts to monitor [14], remove [15], or compensate for them arose. Initially, the three sequencing approaches were evaluated in targeted regions (up to 4 Mbp) [16] where they were compared with Sanger-generated sequences for validation. A recent study compared the accuracy of the SNP calls and the quality of the short-reads from the three platforms in an E. coli sample [17], while another study compared single nucleotide variants from Illumina data with the data from Complete Genomics, another entrant in the sequencing landscape [18].

Here we compare three platforms namely 454/Roche GS FLX, Illumina HiSeq 2000 and ABI SOLiD 3 ECC in their ability to identify the single-nucleotide substitutions in the same human individual. In contrast to previous studies that generated a saturating level of redundant coverage to eliminate low coverage as a factor in SNP calling, we sequenced the individual’s DNA to read-depths that allows for variant detection in each corresponding dataset with sufficient confidence. This allows us to assess the performance and biases of each sequencing platform, as it would affect the completeness of the variant detection in a genome-sequencing project. In this study we show that both Illumina HiSeq 2000 and SOLiD 3 ECC sequencing exhibit variation in coverage with GC content, and report on the probable reasons why certain SNPs are missed by each of the technologies. We also discuss a method to calculate the uniquely mappable regions of a reference genome, which can then be used to filter SNPs and improve the quality of the variant calls, thereby avoiding the generation of false positive variants. This approach is especially useful for 454 GS FLX sequences analyzed using the software Newbler [3], which does not utilize the concept of mapping quality [19].

## Results

### Generation and Alignment of Reads

We sequenced the genomic DNA from a human DNA sample called KB1 [5]; to 10.04 fold coverage using 454 GS FLX sequencer, 58.89 fold coverage using Illumina HiSeq 2000 sequencer and 78.63 fold coverage using SOLiD 3 ECC technology (Table 1). All three platforms are expected to exhibit different error characteristics and therefore should complement one another to yield the most accurate set of human single nucleotide variants. Since the dominant type of error for SOLiD and Illumina reads is substitutions, it is possible to compare the sequences using similar alignment criteria and software. Using the software BWA [20] (version 0.5.9rc), we aligned the SOLiD and Illumina reads to the human reference GRCh37, henceforth referred to as hg19. In contrast to randomly dispersed Illumina and SOLiD sequencing templates, the array-based pyrosequencing technology used by 454 generates sequences with homopolymer errors (indels in runs of the same nucleotide). The assembly/mapping software Newbler aligns these data in a format called flow-space in an attempt to correct these systematic errors associated with pyrosequencing. We used Newbler version 2.3 to align the single-end fragment reads to hg19 with the default parameters. Only the two platforms 454 and Illumina exclude the low-quality reads using filters prior to reporting the final set of reads to the user; a fact that is reflected in a higher alignment rate for these two technologies. In contrast, the SOLiD system uses the alignment to a reference as a mean to determine reads of sufficient quality (Table 1).

**Table 1:** Sequencing and alignment statistics. Coverage is calculated with and without the putative PCR duplicates.

	454	Illumina	SOLiD
Number of reads generated	83,331,227	1,867,073,052	6,905,193,148
Number of bases generated	29,246,232,549	188,349,876,745	397,681,271,500
Read lengths	350 avg. single-end	101 paired-end	50 paired-end, 75 single-end
Number of reads aligned	82,310,265 (98.77%)	1,751,042,389 (93.79%)	4,429,505,837 (64.15%)
Number of bases aligned	28,732,501,185 (98.24%)	168,495,777,999 (89.46%)	224,998,686,646 (56.58%)
Coverage	10.04/9.78 X	58.89/55.06 X	78.63/53.20 X
Duplicate reads	2,211,903	115,528,614	1,216,108,795
Reference bases covered	2,781,827,482	2,858,458,440	2,850,277,778

The number of aligned reads includes the duplicate reads.

### Coverage Distribution and Variation

**Figure 1:**
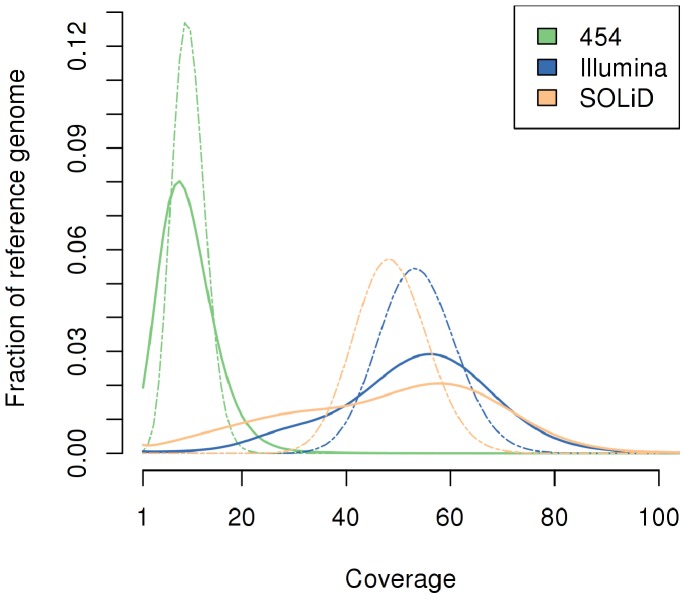
Depth of coverage distribution for the three platforms. The y-axis indicates the fraction of the bases in the reference sequence that has a particular coverage. This does not include secondary alignments and potential PCR duplicates. The dashed lighter curves depict the coverage distribution as calculated using a Poisson model for each sequencing technology.

**Figure 2:**
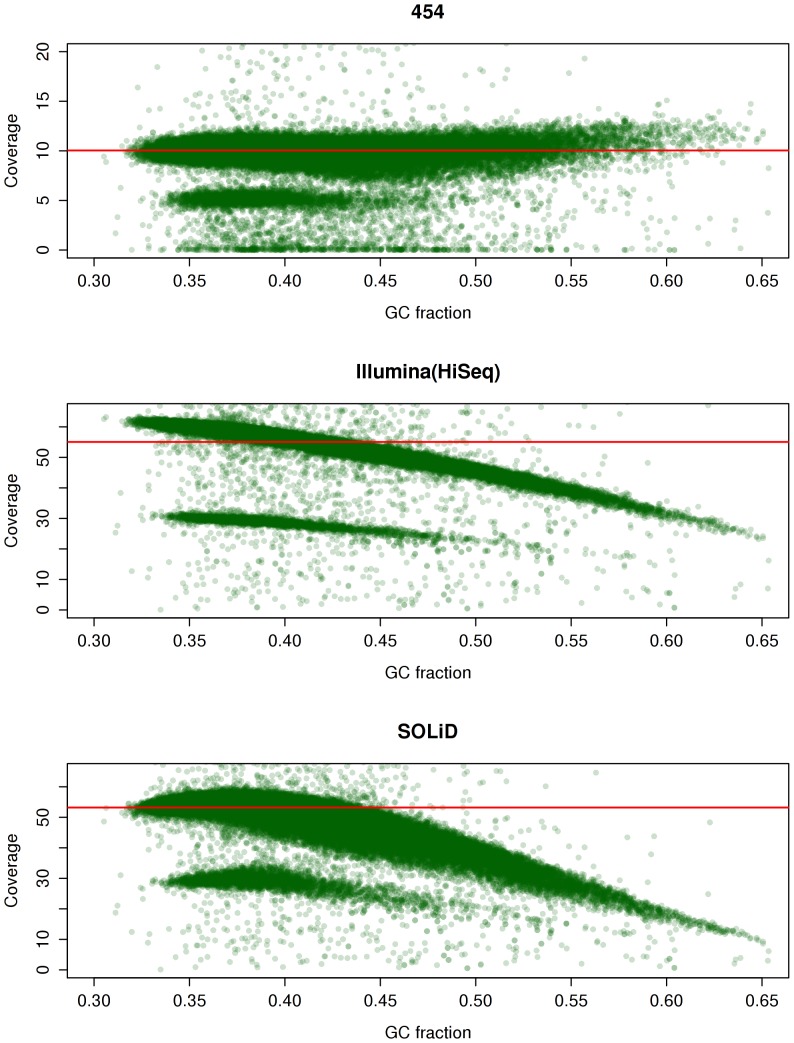
Variation of coverage with GC content in the three sequencing technologies. The red line shows the mean coverage across the whole genome. Each point on the plot reflects the mean coverage and fraction of GC content in 50 kbp non-overlapping window. The y-axis shows the coverage whereas the x-axis shows the fraction of C, G nucleotides in the window. This does not include secondary alignments and potential PCR duplicates.

The depth-of-coverage distributions of the reference genome from data from the three sequencing platforms are shown in Figure 1. The coverage distributions are bimodal (Figure 1), with the two modes that at first appeared to reflect the coverage on the autosomes and the sex chromosomes. However, we found that removing the sex chromosomes from the analysis did not eliminate the bimodal behavior. Since, earlier studies reported a decrease in coverage in A/T rich regions with Illumina sequencing [21], we investigated the correlation between the GC content and coverage for the three platforms, for potentially influencing bimodal behavior. Figure 2 shows a significant variation in coverage with GC content; coverage by Illumina and SOLiD sequences is notably lower in G/C rich regions. This variation with GC content, along with the expected haploid coverage on sex chromosomes, explains the observed bimodal behavior of these distributions. Despite sharing the emulsion PCR approach in the sequencing template preparation with the SOLiD 3 system, only the Roche/454 FLX sequencing chemistry seemed to be immune to this bias, as is demonstrated with only a minor correlation between GC content and coverage for 454 reads (Figure 2). The behavior exhibited by Illumina HiSeq sequences is in stark contrast to the behavior of GA II reads, which exhibit a lower coverage in A/T rich regions.

### Detection of SNPs and Indels

**Figure 3:**
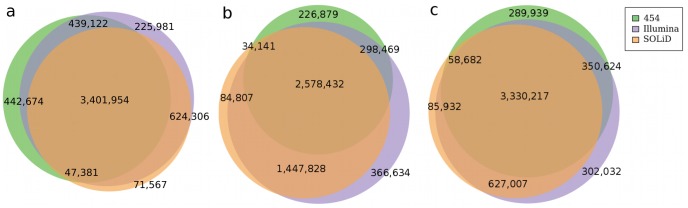
Venn diagram showing the overlap in the SNP calls made using data from the three sequencing technologies. We display the sizes of each of the seven categories of overlaps among the variant calls in the three technologies. (a) depicts the overlaps when all substitution calls are used, (b) depicts the overlaps when all calls from Illumina and SOLiD are used but only the high-confidence subset of the 454 dataset is used, and (c) depicts the overlaps when only the variants in the uniquely alignable regions of the reference sequence are used.

A large portion of the genomic regions requires local realignments due to the presence of indels in the sequenced genome when compared to the reference genome. Indels can lead to alignment artifacts where a lot of bases around the indel do not agree with the reference and can masquerade as SNPs. We used the realignment tool in GATK [22] version 1.2–29 to realign the sequences from the Illumina and SOLiD dataset; followed by use of SAMtools version 0.1.16 to call variants. As described in the previous section, Newbler handles the 454 data in flow-space and we used Newbler version 2.3 to call variants in it (See Methods). While the three sequencing approaches resulted in a similar number of single substitution variants, namely 4,331,131 variants for the 454 data, 4,691,363 variants using the Illumina data, and 4,145,208 variants using the SOLiD sequences, the combination resulted in a total of 5,252,985 potential variant locations. However, only a common set of 3,401,954 variant locations was shared between all three technologies, whereas only one or two of the platforms supported the remaining 1,851,031 locations (Figure 3a). As for indels, we found 614,794 indels using the 454 data, 554,138 small indels using the Illumina data and 303,937 potential indels using the SOLiD data.

### Reasons for Failure of Detection of Substitutions

The 1,851,031 discrepant locations between 454 FLX, Illumina HiSeq and SOLiD 3 sequences allow for the study the false positive and the false negative rates for the obtained variant calls. Considering the SNP calls from each platform as independent evidence, we use the locations where two platforms agree to study the false negatives for the third one. This allows us to quantify and understand the reasons why this SNP was not called in the dataset sequenced using the third platform. Similarly, at the locations where only one platform calls a variant, we identify and study the false positives for that platform, barring exceptions that are explained later in the text.

The reasons why a SNP is not detected by one sequencing technology, whereas it is reported by another, can be broadly divided into three categories:

- **Issues related to coverage**: These can be further subdivided into complete lack of coverage, low coverage (which is not enough to call a SNP based on predefined criteria), and higher-than-expected coverage (based on a model used to separate SNPs from structural variants and assembly errors) at the candidate location.
- **Issues with the alternate allele**: Most software tools (including SAMtools and Newbler) require observing the alternate allele at least twice or more, before they consider the location as a potential variant. These can be further subdivided into instances where the alternate allele is not seen at all and others, when the alternate allele is not seen a sufficient number of times.
- **Issues with the variant calling**: These refer to the situations where the alternate allele is seen a requisite number of times, but the SNP is not called due to other reasons. These reasons may include proximity to many other SNPs, proximity to a high quality indel, existence in a non-uniquely alignable region, and a huge deviation from the expected diploid behavior of the sample for the data aligned using BWA. For the reads aligned using Newbler, the reasons include the location being in a non-uniquely alignable region and other alignment errors that arise due to the unique error-profile of the 454 reads.

**Figure 4:**
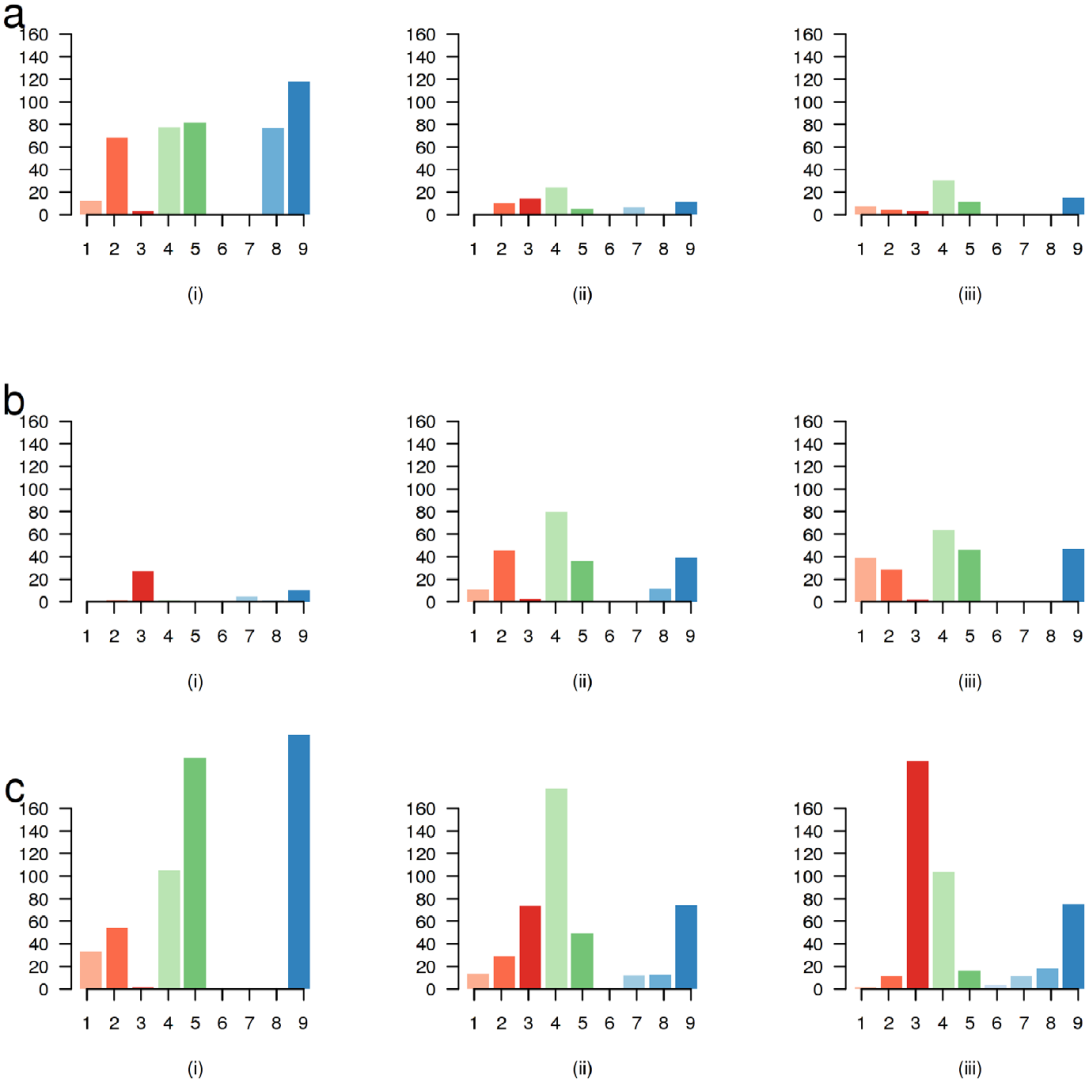
Discrepant SNP calls from each platform. The categories on the x-axis are (1) no coverage at location (2) not enough coverage at location (3) more than expected coverage (4) alternate allele not seen (5) alternate allele seen just once (6) too many SNPs around location (7) close to a high-quality indel (8) low RMS mapping quality (9) low SNP quality. The y-axis depicts the number of locations (frequency) in each category. a) Comparison of SOLiD generated sequences with other sequences based on SNP calls and alignments. (i) SNPs called using 454 and Illumina sequences but not called using SOLiD reads. (ii) SNPs called only by SOLiD sequences. We investigate why they were not called using Illumina alignments. (iii) SNPs called only by SOLiD sequences. We investigate why they were not called using 454 alignments. b) Comparison of Illumina generated sequences with other sequences based on SNP calls and alignments. (i) SNPs called using 454 and SOLiD reads but not called using Illumina reads. (ii) SNPs called only by Illumina sequences. We investigate why they were not called using SOLiD alignments. (iii) SNPs called only by SOLiD sequences. We investigate why they were not called using 454 alignments. c) Comparison of 454 generated sequences with other sequences based on SNP calls and alignments. (i) SNPs called using SOLiD and Illumina reads but not called using 454 reads. (ii) SNPs called only by 454 sequences. We investigate why they were not called using SOLiD alignments. (iii) SNPs called only by 454 sequences. We investigate why they were not called using Illumina alignments.

We investigated the alignments at the 439,122 locations that were called as putative variants by using 454 and Illumina sequences, but not using SOLiD sequences (Figure 4a i). We assigned each location to a particular category based on the reason why it was not called a SNP. We found that the variant allele was observed in the SOLiD reads in 64% of these cases, but the SNP was filtered away for various reasons. 27% of the locations were filtered away due to a low SNP quality (defined as the Phred-scaled likelihood that the called genotype is identical to the reference), 18% of them were filtered away due to a low RMS (root mean square) mapping quality (reflecting the limitation of shorter reads) and another 19% were filtered away as the variant allele was not seen enough number of times. Coverage related issues (no coverage, too little coverage or more than expected coverage) were responsible for another 19% of the locations. The alternate allele was not seen at all, despite adequate coverage at the site, for the remaining 17% locations.

For the 71,567 locations that were called using the SOLiD sequences (but not by others), we looked at the alignments for both the 454 dataset and the Illumina datasets. At about 15% of these locations (Figure 4a ii), the alternate allele was seen just once in the 454 dataset and at about another 16% of them, the coverage of 454 reads was not enough to call a SNP. For another 21% of the locations the SNP was not called by Newbler, even though the allele was seen multiple times in the pairwise alignments between the reference and the 454 reads, with most of them being associated with homopolymer errors. On the other hand at 25% of these locations the SNP was seen in the Illumina dataset (Figure 4a iii), but it was filtered away due to a lower SNP quality (15%), or because lower mapping quality (9%). Another 14% of these locations did not have sufficient coverage with Illumina reads to be considered in SNP calling. Considering the locations where both 454 and Illumina had little, no, or higher than expected coverage, and where the alternate allele was seen at least once in either 454 or Illumina dataset as true SNPs, we expect 14,707 of the 71,567 locations to be false-positives for the SOLiD calls.

When we looked at the 47,381 locations that were called a SNP using 454 and SOLiD reads, we found that primary reason (at 60% of the locations) these were not called a SNP with Illumina reads had to do with the coverage (Figure 4b i). 57% of the locations were in regions where the coverage was more than expected (signaling a putative structural variant), whereas there was little of no coverage for the remaining 3%. We used a Poisson distribution with the same mean value to calculate the coverage threshold to filter variants, but this data suggests that a gamma distribution with more weight on more tails is probably a better model for Illumina data. The second largest contributor was low SNP quality (22% of the locations), which is the result of an observed deviation from the expectation that both allele should be seen approximately the same number of times on a heterozygous location.

We found 225,981 locations that were called as putative variants using Illumina reads only. Looking at the alignments for the SOLiD reads at those locations (Figure 4b ii), we found that for 22% of them we saw the alternate allele a sufficient number of times, but it was filtered away either due to low RMS mapping quality or a low SNP quality. Another 16% of the locations were filtered away as the variant was not seen sufficient number of times. The alternate allele was not seen at all for 35% of the locations and the coverage was deemed insufficient for the remaining 27% of the locations. This was very different from 454 (Figure 4b iii), where the alternate allele was not seen at all for about 28% of the locations, only seen once for about 20% of the locations, and for another 21% the allele was seen multiple times but was not called as a variant by Newbler. Using the same criterion as for SOLiD data, we expect 31,696 of the calls to be false-positives for the Illumina data.

Among the 624,306 locations that were called a SNP using both Illumina and SOLiD reads, but not using 454 reads, 225,443 (36%) had the alternate allele multiple times in the pairwise alignments of the reads with the reference (Figure 4c i). However, they were not flagged as variants in the later stages of processing, with most of them being associated with homopolymer errors. There were another 205,060 SNPs (33%) where we saw the alternate allele once, not twice or more as required by Newbler to call it a SNP. As for the 442,674 locations that were only called as variants using 454 sequences, SOLiD (Figure 4c ii) and Illumina (Figure 4c iii) did not call 19% of them due to low RMS mapping quality or low SNP quality. 45% of the variant locations were not considered in Illumina reads as the coverage exceeded the expectations, and another 23% were not called as the variant was not seen enough times. In contrast, only 17% of the locations had more than expected coverage in SOLiD reads, but 40% of the variants were not called as the variant allele was not seen enough times. We expect 75,695 calls to be false-positives for sequences generated using 454 instruments for this sample using the same criterion as that used for Illumina and SOLiD. However a large proportion of these putative false-positive calls were in locations where both Illumina and SOLiD saw the alternate allele multiple times, but the SNP was filtered away as the depth-coverage at that location was higher than expected. This could be an artifact of our SNP-calling pipeline, which used Poisson distributions to set the coverage thresholds for the SNP calls, or a consequence of structural variants present in the target genome. This also means that false-positives for 454 sequences are indicative of certain biases in Illumina and SOLiD reads along with errors in the 454 reads.

### 454 Data and Newbler-specific Variant Calling Variation

Newbler labels a subset of its variant calls as “high-confidence”. These calls require more evidence than that required for the calls used in the analysis summarized above. We therefore decided to use the high-confidence subset of the 454 FLX calls to investigate whether this increases the concordance rate of 454 data with the other two technologies. Newbler called 3,137,921 substitutions when the high-confidence subset was used, resulting in a reduction from 442,674 454-specific calls (with default parameters) to now only 226,879. The more stringent setting, however, significantly increased the number of SNPs that were called by Illumina and SOLiD, but not by 454, to 1,447,828. It also decreased the number of SNPs called by all three technologies to 2,578,432 (Fig. 3b). Therefore, even though using the high-confidence subset of SNP calls seems to reduce the false-positive rate of calls (as evident by a decrease in the number of calls made only by 454 reads), it significantly increases the false-negative rate for variant calls.

BWA and many other aligners assign a mapping quality to each read, which is a scaled error probability that the alignment for the read is wrong. A read with a mapping quality of zero does not contribute to SNP calls. Newbler on the other hand aligns the reads to the reference and if it finds more than one alignment that passes all its alignment thresholds, then it tags the read as a “repeat”, and that read is not used in SNP calling. Both methods attempt to reduce the false-positive rate of SNP calls, while attempting to use as much of the data as possible.

We normally post-process the SNP calls to restrict the calls to the uniquely alignable regions of the reference genome, for the reads of a certain length and error profile. This method was used in Schuster et al. 2010 [5] to call the variants and explicitly discussed in Koehler et al. 2010 [23], which termed the uniquely alignable region of the reference as the “uniqueome”. We calculated the uniquely mappable region for 454 reads of average length 350 bp, 76 bp paired-end Illumina reads and 75 bp single-end/50 bp paired-end reads for SOLiD reads, and then filtered to throw away the SNP calls that did not lie in these regions. The results are shown in Figure 3c, where we see a substantial decrease in the calls made only by 454 sequencing, a marginal decrease in the number of calls made only by SOLiD sequencing, and an increase in Illumina only calls.

Our study highlights some of the issues encountered when these technologies are used for whole-genome sequencing of samples. We realize that the results of our analyses reflect a cumulative effect of library preparation, base callers, sequencing bias, alignment tools and variant callers. All platforms have their own biases, and use of more than one technology can be used to overcome the limitations that are posed by a single sequencing technology.

### Comparison of Indels

Insertions and deletions are harder to call and analyse, compared to single nucleotide substitutions. Calculation of indel boundaries requires computationally expensive local realignments, and even then the exact start and stop of an indel can vary between algorithms and samples, making any comparison based on coordinates difficult. We had fragment reads from 454, and paired-end reads from Illumina and SOLiD (with different insert length distributions); each technology is best suited for different lengths and ranges of indels making any meaningful comparison difficult. Prior report on indels in whole-genome sequences have varied greatly; from 135,262 in the Han Chinese genome, to 823,396 indels in the Venter genome, showing the limitations faced in calling these variants.

Newbler called 614,794 indels in KB1 with the 454 data, 225,980 of which were homozygous and 282,909 of them were tagged as high confidence. Intersection of indel intervals in dbSNP 132 with these indel calls results in 263,262 (42.82%) overlaps and another 67,895 (11.04%) indels are found with +/−10 bp of the indels in dbSNP. We called 554,138 indels using the Illumina data, and 303,937 indels with the SOLiD data. 440,514 (79.49%) of the indels called using the Illumina data and 255,232 (83.97%) of the indels called using the SOLiD data were also found in dbSNP132. These numbers increased to 456,328 (82.35%) and 259,333 (85.32%) when an overlap +/−10 bp around indels was allowed.

In terms of overlap between the technologies, out of the possible 842,281 intervals, 177,772 of the indels were called using the sequences from all the technologies. 223,029 of them were supported by 2 of the 3 technologies, whereas the remaining had support from just one of them.

### Experimental Validation of SNPs

**Figure 5:**
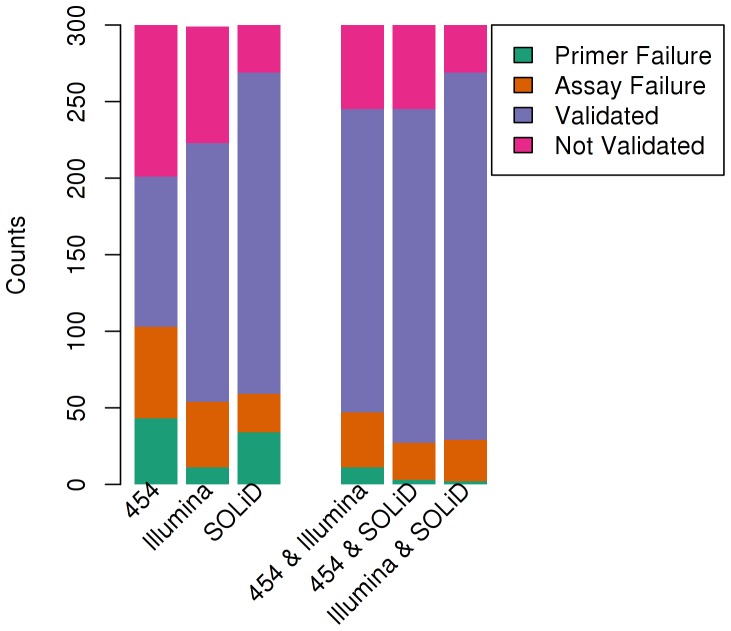
SNP Validation using Mass spectroscopy. Validation of 300 putative SNP locations from each of the six sets of SNP calls in Figure 3a, where not all three technologies agree on the computed genotype. The categories on x-axis are “454” (SNPs called by 454 only), “Illumina” (SNPs called by Illumina only), “SOLiD” (SNPs called by SOLiD only), “454 & Illumina” (SNPs called by 454 and Illumina), “454 & SOLiD” (SNPs called by 454 and SOLiD), “Illumina & SOLiD” (SNPs called by Illumina and SOLiD). The color categories include “Primer Failure” (Primer extension failure), “Assay Failure” (Assay Failure), “Validated” and “Not Validated”.

We randomly selected 300 SNPs from each of the six sets in Figure 3a, i.e., where at least one of the three platforms (454 GS FLX, Illumina HiSeq 2000, SOLiD 3) disagreed on the computed genotype. For this purpose a sequence interval of 50 bp flanking a potential variant on each side was used for primer design using the MassARRAY® Assay Design Software (Sequenom, Inc.). These SNPs were assayed using mass spectrometry based genotyping technology and the results are shown in Figure 5. The validation rate was 50% for variants supported only by 454, 69% for variants only supported by Illumina, and 87% for the ones supported only by SOLiD. The validation rate for variants supported by more than one platform was higher when compared to the rate from the individual platforms, e.g. the validation rate for variants supported by 454 and Illumina was 78% whereas the validation rate for variants supported only by 454 was 50% and the validation rate for variants supported only by Illumina was 69%. The validation rate for SNPs called using 454 and SOLiD was 80% and it was 89% for variants called by Illumina and SOLiD. The longer reads generated by 454 resulted in some SNPs with 50 bp flanks (as required by the Sequenom assay) in repeat regions that have a higher dimer/hairpin potential when compared to the flanks for SNPs in the other two platforms. As a result, we saw significantly more primer design and assay failures for the 454 derived SNPs when compared to SNPs in the two short read technologies. Furthermore, the number of assays and primers that failed was also notably higher for locations that had been called by only one of the sequencing technologies.

## Discussion

With the ultimate goal to sequence and analyze a human genome as fast and cost-effective as possible, it is highly desirable to simplify sampling and library preparation steps, but also to carry out DNA-sequencing on a single technology platform. In fact, with the availability of a high quality reference genome, re-sequencing with “low-cost per base” technologies has been at the center of recent efforts of most academic and commercial sequencing endeavors. However, with hundreds of human genomes currently being deciphered, the question of completeness relative to today’s technological possibilities has become secondary to other pursuits. Faced with increasing evidence that human genomics does not provide allelic association to critical human phenotypes at predicted rates, the question is being raised whether missed genetic variants might be causal due to unmapped regions of the genome. Further, understanding interactions of so far unrecognized genetic variations with small molecule drugs is of increasing interest to pharmaceutical and diagnostic industry. Our analysis on the false negative rate of SNP identification suggests that a significant number of variants are not reported using a single sequencing platform, limiting insights which could enable a more complete understanding of the human genome. On the other hand, false positives from a single sequencing technology lead to a continuous degradation of the quality of SNP databases via the inclusion of non-existing genetic variants. Using a non-sequencing approach we report that the validation of SNP calls is significantly improved for variants that are reported using more than a single sequencing platform.

We realize that every lab and sequencing platform has a “best practice” protocol from sample-prep to the bioinformatics pipeline, and that all the steps heavily influence any conclusion that one attempts to derive in such an experiment. In this study we show evidence that each of the three applied sequencing technologies contributes, at the respective coverage of each dataset, an additional unique 71,000 to 443,000 variants (1.4–8.9%) of the total of 5 million found in the human individual KB1. Remarkably, at least 1.4% of these technology-dependent variants would have gone unnoticed, even if the genome were sequenced on two of the three platforms. Furthermore as evident using Sequenom mass spectrometry, the validation rates for variants using two platforms are improved by more than 10% over the validation rates for the individual platforms. It therefore seems highly beneficial to sequence at least reference genomes from each geographic region by the multi-platform approach presented here. A larger number of multi-platform human reference genomes would not only minimize systematic technological biases, but also reduce ethnical biases from today’s human genome reference sequence.

In this comparative study we looked at nine factors that affect the ability of a sequencing platform to accurately call variants in the human genome (see Figure 4 a–c). That include coverage related issues, allele related issues where the alternate allele was not seen multiple times and SNP related issues where the SNPs were filtered away in an attempt to reduce the false-positives. Among all the factors impacting the probability of a variant to be called, one of the most important one is the unbiased distribution of reads across a genome. As shown in Figure 2, the Roche/454 data is much more uniformly aligned, independent from the GC content of the genome than the short read platforms, resulting in comparable number of variant calls despite a much lower overall coverage. This trend however is counteracted by the 454 Roche specific error model that is prone to inaccurately assess the length of a given homopolymer, leading to potential false-positive SNPs calls, as shown by calls made only by 454 sequencing (Figure 3a). In addition to the platform specific errors, systematic errors are introduced through the varying algorithms and parameters used in the process of variant calling. This becomes apparent when the Roche/454 dataset is reanalyzed to include only the “high-confidence” variants, which reduces the total number of detected Newbler SNPs by 21%. The Roche/454 specific SNPs are reduced this way by more than 51% (226,879 calls, down from 442,674) (Figure 3b), however at the cost of increasing potential false negative prediction, as is apparent in a largely increased number of calls that are seen only by the Illumina and SOLiD platform (1,447,828 calls, up from 624,306). In order to avoid the conflict of Newbler’s two modes of that either over-predicts false-negative or under-predicts true positive variants, we calculated the uniquely mappable region of the human genome for each dataset specific on the platform’s read length (Roche/454 length 350 bp, Illumina 76/101 bp paired-end reads and SOLiD 75 bp single-end, 50 bp paired-end reads). This approach is highly successful in combining the sensitivity of the variant detection for the Roche/454 data, while significantly reducing the rate of false negative prediction, as shown in Figure 3c. In our approach, the total number of variants agreed upon by all three platforms remains by ∼3.3 million, while the number of Roche/454 specific variants is lowered to 302,032.

Interestingly, the lower GC bias as seen in 454 reads is not observed for the Life Technologies/SOLiD platform, despite the fact it also uses emulsion-PCR for template amplification. The performance of the SOLiD system was greatly aided in this comparison by its much higher coverage when compared to the 454 or Illumina data set.

An ideal setup for a high quality human or other mammalian genome project therefore would include all three sequencing platforms and thus allow for the generation of the most complete SNP sets and genome sequences. While we find that the three technology platforms complement one another, efforts are underway to improve currently used chemistries to combine advantages from multiple platforms into a single one.

## Methods

### Sequencing

#### 454 Sequencing on the GS FLX

The protocols and details of library construction and sequencing on four Roche/454 GS FLX instruments using Titanium chemistry, for a total of 72 runs have been previously described in Schuster et al, 2010.

#### Illumina Sequencing on the HiSeq 2000

The KB1 library was prepared from genomic DNA using the Multiplexing Sample Preparation Oligonucleotide Kit from Illumina according to manufacturers instructions. The library was subjected to 5 cycles of PCR enrichment prior to cluster generation and then sequenced on the HiSeq 2000 according to manufacturers instructions.

#### SOLiD sequencing using SOLiD 3 ECC platform

SOLiD sequencing of KB1 was performed using recent developments in ligation-based sequencing. Specifically, improvements in paired-end sequencing have allowed us to sequence longer read lengths in both the forward (75 bp) and reverse direction (30 bp). The longer paired end reads were combined with a mate pair (2×50, 1.5 kb insert size) sequencing approach to achieve ∼60× paired coverage. In addition, an increase in accuracy was obtained by using Exact Call Chemistry (ECC) for mate pair sequencing, as well as the 75 base pair forward tag.

ECC is based on standard “error correcting codes” commonly used in modern communication and data storage systems. This approach works by transforming data and augmenting it with redundancy to make it more resistant to measurement error. SOLiD and its ligation-based sequencing approach has the unique ability to use ECC by applying an additional, second sequencing probe set and repeating a sequencing primer for redundancy (Figure 5). This reduces measurement error and improves accuracy with minimal impact on sequencing time. Furthermore, ECC allows one to decode SOLiD sequencing data directly to base space without using a reference by using Bayesian inference. All of the analysis on the data was performed in base space and more details about the involved chemistry can be found at http://www3.appliedbiosystems.com/cms/groups/global_marketing_group/documents/generaldocuments/cms_091372.pdf.

### Alignment of Reads

#### 454

We used Newbler version 2.3 to align the 454 reads to the human reference genome (hg19) using the default parameters.

#### Illumina

We aligned the Illumina reads to the human reference genome (hg19) using BWA version 0.5.9rc, allowing up to 2 differences for reads of length 36 bp, up to 4 differences for reads of length 76 bp and up to 5 differences for reads of length 101 bp. The base quality deteriorates towards the 3′ end of the read, so we ran BWA with an option ‘–q 20′ to allow trimming of the read down to 35 bp.

#### SOLiD

We aligned the SOLiD reads to the human reference genome (hg19) using BWA version 0.5.9rc, allowing up to 3 differences in reads of length 50 bp and up to 4 differences in reads of length 76 bp. All the other parameters and arguments used were the same as that used for alignment of the Illumina reads.

### Coverage

Coverage from the three technologies is defined as:

C = (number of distinct bases aligned)/(number of non-N bases in hg19 = 2,861,343,702).

### Removal of Duplicate Reads

We used the MarkDuplicates tool offered as part of the Picard command-line suite (http://picard.sourceforge.net) to mark the duplicate reads in the Illumina and SOLiD datasets. Newbler takes care of the duplicate reads internally by requiring that variants be confirmed from non-duplicate reads. However, we did calculate the number of duplicate reads in the 454 dataset (Table 1) to compare them to Illumina and SOLiD, by calculating the number of reads that had the same alignment coordinates on the reference.

### Variant Calling

In previous studies [16], Poisson distribution has been assumed as the model of depth coverage and we used it to set the coverage thresholds to filter SNPs.

#### 454

We used Newbler version 2.3 to align and call SNPs from the 454 reads. The SNPs (available in the file 454AllDiffs.txt produced by Newbler) were analysed and were kept if the coverage at the SNP location was between 2 and 30. We deemed the SNPs as homozygous if more than 80% of the reads supported the alternate allele. We filtered to only keep the homozygous SNPs from the sex chromosomes and the mitochondria.

#### Illumina

We used SAMtools version 0.1.16 to call the variants in the Illumina reads. We required a minimum coverage of 4, a maximum coverage of 60 and a minimum quality of 20 for the SNPs and indels that were found to be on the autosomes. We reduced the maximum coverage requirement to 45 for the sex chromosomes and increased it to 10,000 for the mitochondrial DNA. Only homozygous SNP and indels calls were kept from the sex chromosomes and mtDNA.

#### SOLiD

We used SAMtools version 0.1.16 to call the variants in the Illumina reads. We required a minimum coverage of 4, a maximum coverage of 100 and minimum quality of 20 for the SOLiD reads that mapped to the autosomes. The maximum coverage limit was reduced to 60 for the sex chromosomes, and increased to 10,000 for the mitochondrial DNA and only homozygous SNP and indel calls were kept from these chromosomes.

### Variation with GC Content

We calculated the mean coverage and the GC content in non-overlapping windows of 50 kbps and that information was used to plot Figure 2.

### Selection of SNPs for Validation

We collated the SNPs belonging to each of the six categories in Figure 5, where the three technologies did not agree on the call. We removed the SNPs where the 50 bp flanks for the SNPs aligned to more than one location on hg19 with greater than 95% identity. We randomly selected 300 SNPs from each of the categories for validation.

### Single Copy Intervals in hg19

We used a “self-masking” process to identify the regions in the reference genome where reads should align uniquely. The process breaks the reference genome into smaller fragments of length equal to the length of the reads, and adjacent fragments overlap each other by half the read length. These fragments are aligned to each reference chromosome, with alignment parameters selected to allow differences with a certain mutation rate. Any reference position appearing in no more than two alignments is considered uniquely mappable, since we only expect it to align to the two fragments that include it. We used LASTZ [24] and utilized the dynamic masking option to mask the reference genome. Initially the reference sequence is marked entirely as non-masked, and counters associated with each base are set to zero. Scoring parameters are set to reflect those that will be used later to map reads. As alignments are found, the corresponding counter is incremented for every base in the alignment. Whenever a base’s counter exceeds 2, that base is soft-masked and removed from the alignment seeding tables. This latter action prevents highly repetitive regions from overwhelming the computational process.

### Sequenom Genotyping

#### Whole Genome Amplification (WGA)

We amplified genomic DNA samples using the REPLI-g Whole Genome Amplification Midi Kit (Qiagen, Valencia CA). Amplified DNA was cleaned up as per manufacturer’s recommendation.

#### Sequenom validation

The predicted SNPs were validated on the Sequenom Mass Spectrometry platform using the iPLEX Gold Chemistry (Sequenom, San Diego CA). Primers for validating the SNPs were made using iPLEX Assay Design 3.0 software (Sequenom, San Diego, CA). The software creates a pair of primers that will allow amplification of an approximately 100 base pair PCR product that encompasses the region where the SNP of interest occurs. It also creates an extension primer that is complementary to the region of interest and extends up to the penultimate base right before the SNP position that is being interrogated. The PCR and extension primers used in this study are listed in Table S1. The primers used in the study were synthesized at Integrated DNA technologies in Iowa. PCR reactions with the appropriate template DNA and 100 nM of each PCR primer was set up and brought to 94°C and held for 2 minutes at 94°C followed by 35 cycles of 94°C for 15 sec, 56°C for 30 sec and 70°C for 1 min. A final extension setup at 70°C for 8 min concluded the PCR. Excess primers and unincorporated deoxynucleotides were removed from PCR reactions by adding 0.3 units of shrimp alkaline phosphatase (Sequenom, San Diego, CA), 0.3 to each reaction and incubating at 37°C for 40 min. The shrimp alkaline phosphatase was heat inactivated for 5 min at 85°C. The PCR reactions products were then subject to single base pair extension using the extension primer Thermosequenase and iPLEX nucleotides (Sequenom, San Diego, CA). Extension primers were used at a final concentration of 0.2 µM in a 10 µl reaction. Extension reactions were initially incubated at 94°C for 30 sec followed by 40 cycles of 94°C for 5 sec, 5 cycles of 54°C for 5 sec and 80°C for 5 sec. After this, primer extension reaction products were desalted with SpectroCLEAN resin (Sequenom, San Diego, CA). Ten nanoliters of the extension reaction was dispensed on a 384-format SpectroCHIP (Sequenom) prespotted with 3-hydroxypicolinic acid using a MassARRAY nanodispenser (Sequenom, San Diego, CA). Data was collected using a matrix-assisted laser desorption ionization/time of flight mass spectrometer (Sequenom, San Diego, CA). Primer extension data was analysed using MassARRAY Typer 3.4 software (Sequenom, San Diego, CA).

## Supporting Information

Table S1 **The putative allele, the flanking sequence in the genome, PCR and extension primers used in this study are listed in this spreadsheet.** The sheet “Only 454” lists the details for locations that were only called using 454 generated sequences, “Only Illumina” refers to the details for locations that were only called by sequences generated using Illumina HiSeq 2000 sequencing, and “Only SOLiD” refers to the details for the locations that were only called by sequences generated using SOLiD 3 ECC technology. “454 and Illumina”, “454 and SOLiD”, and “Illumina and SOLiD” refer to details for locations that were called by only two of the three sequencing platforms that were used in this study.
(XLSX)
